# Identification of Natural Resistance Mediated by Recognition of *Phytophthora infestans* Effector Gene *Avr3a^EM^* in Potato

**DOI:** 10.3389/fpls.2020.00919

**Published:** 2020-06-19

**Authors:** Ahmed S. M. Elnahal, Jinyang Li, Xiaoxia Wang, Chenyao Zhou, Guohong Wen, Jian Wang, Hannele Lindqvist-Kreuze, Yuling Meng, Weixing Shan

**Affiliations:** ^1^State Key Laboratory of Crop Stress Biology for Arid Areas and College of Plant Protection, Northwest A&F University, Yangling, China; ^2^Plant Pathology Department, Faculty of Agriculture, Zagazig University, Zagazig, Egypt; ^3^Institute of Potato Research, Gansu Academy of Agricultural Sciences, Lanzhou, China; ^4^Institute of Biotechnology, Qinghai Academy of Agricultural Sciences, Xining, China; ^5^International Potato Center, Lima, Peru; ^6^State Key Laboratory of Crop Stress Biology for Arid Areas and College of Agronomy, Northwest A&F University, Yangling, China

**Keywords:** potato late blight, *Phytophthora infestans*, *PiAvr3a^EM^*, Qingshu9, Longshu7, *Agrobacterium tumefaciens*, RXLR effectors, hypersensitive response

## Abstract

Late blight is considered the most renowned devastating potato disease worldwide. Resistance gene (*R*)-based resistance to late blight is the most effective method to inhibit infection by the causal agent *Phytophthora infestans*. However, the limited availability of resistant potato varieties and the rapid loss of *R* resistance, caused by *P. infestans* virulence variability, make disease control rely on fungicide application. We employed an *Agrobacterium tumefaciens*-mediated transient gene expression assay and effector biology approach to understand late blight resistance of Chinese varieties that showed years of promising field performance. We are particularly interested in *PiAvr3a^EM^*, the most common virulent allele of *PiAvr3a^KI^* that triggers a *R3a*-mediated hypersensitive response (HR) and late blight resistance. Through our significantly improved *A. tumefaciens*-mediated transient gene expression assay in potato using cultured seedlings, we characterized two dominant potato varieties, Qingshu9 and Longshu7, in China by transient expression of *P. infestans* effector genes. Transient expression of 10 known avirulence genes showed that *PiAvr4* and *PiAvr8 (PiAvrsmira2)* could induce HR in Qingshu9, and *PiAvrvnt1.1* in Longshu7, respectively. Our study also indicated that *PiAvr3a^EM^* is recognized by these two potato varieties, and is likely involved in their significant field performance of late blight resistance. The identification of natural resistance mediated by *PiAvr3a^EM^* recognition in Qingshu9 and Longshu7 will facilitate breeding for improved potato resistance against *P. infestans*.

## Introduction

Potato (*Solanum tuberosum* L.) is regarded as the fourth-largest food crop and the main non-cereal crop worldwide which is influenced by the destructive and notorious late blight disease (Aguilera-Galvez et al., 2018). *Phytophthora infestans* is the causative agent that can destroy all potato parts, including leaves, stems and tubers (Fry, 2008), and is the main threat to potato production and responsible for 16% of yield losses globally (Haverkort et al., 2016). Similar to other crops, disease management using resistant varieties is one of the most effective strategies, environmentally and economically, to control late blight disease (Fry, 2016). Plant immunity is activated by detecting conserved microbial molecules, microbe (pathogen)-associated molecular patterns (MAMPs or PAMPs), known as pattern-triggered immunity (PTI), as well as by detecting the pathogen effectors, known as effector-triggered immunity (ETI) (Jones and Dangl, 2006). Plant pathogens can successfully colonize plant hosts by delivering effector proteins that repress host immunity and increase disease severity (Turnbull et al., 2017). In turn, few effectors might be recognized by the correspondent resistance (R) proteins, triggering a rapid immune response known as effector-triggered immunity (ETI), which often leads to an hypersensitive response (HR) cell death (Jones and Dangl, 2006; Turnbull et al., 2017).

To achieve effective control of late blight, potato breeders have to adopt novel techniques and strategies in various aspects such as detection and identification of new *Rpi* genes, their introgression and field application (Vleeshouwers and Oliver, 2014). Great efforts were made at the beginning of the last century to introgress *Rpi* genes into potato varieties from the wild Mexican species *Solanum demissum* to provide resistance to *P. infestans* in the cultivated potato *S. tuberosum*. This also led to the development of differential potato lines with 11 distinct recognition specificities, called *R1-R11* (Black et al., 1953; Malcolmson and Black, 1966). Many *R* genes (*Rpi*) have been identified, cloned and some of them were introgressed into potato cultivars from wild Mexican *Solanum* species (Goodwin et al., 1992; Grunwald and Flier, 2005; Goss et al., 2014), including *R1–R11*, *R3a*, *R3b*, *R9a* and *Rpi-demf1* from *S. demissum* (Ballvora et al., 2002; Huang et al., 2005; Lokossou et al., 2009; Jo et al., 2015), *Rpi-blb1*(*RB*), *Rpi-blb2*, *Rpi-blb3*, *Rpi-abpt* and *Rpi-bt1* from *S. bulbocastanum* (Song et al., 2003; Van der Vossen et al., 2003; Park et al., 2005; Van der Vossen et al., 2005; Lokossou et al., 2009), *Rpi-sto1,2*, *Rpi-pta1,2*, and *Rpi-plt1* from *S. stoloniferum* (Vleeshouwers et al., 2008; Wang et al., 2008; Champouret et al., 2009), *Rpi-amr3* from *S. americanum* (Witek et al., 2016), *Rpi-mch1* from *S. michoacanum* (Sliwka et al., 2012a), and *Rpi1* from *S. pinnatisectum* (Kuhl et al., 2001; Lokossou et al., 2010). Additionally, further *R* genes have been identified in another center of genetic diversity of tuber-bearing *Solanum*, the Andean region in South America, such as *Rpi-vnt1* from *S. venturii* (Foster et al., 2009; Pel et al., 2009), *Rpi-mcq1* from *S. mochiquense* (Smilde et al., 2005), *Rpi-ber* from *S. berthaultii* (Rauscher et al., 2006), *Rpi-mcd1* from *S. microdontum* (Tan et al., 2008), *Rpi-pcs* from *S. paucissectum* (Villamon et al., 2005), *Rpi-cap1* from *S. capsicibaccatum* (Jacobs et al., 2010), *Rpi-rzc1* from *S. ruiz-ceballosii* (syn. *S. sparsipilum*) (Śliwka et al., 2012b; Brylińska et al., 2015) and *Rpi-chc1* from *S. chacoense* (Zhu et al., 2015).

Much attention has been paid to RXLR effectors since the cloned avirulence protein (Avr) of oomycete pathogens belong to this type of effector, such as *P*. *sojae Avr1b* (Shan et al., 2004), *Hyaloperonospora arabidopsidis ATR13* (Allen et al., 2004) and *ATR1* (Rehmany et al., 2005), and *P. infestans PiAvr3a* (Armstrong et al., 2005). So far, over 10 *Avr* genes, identified by 10 cognate-recognition *Rpi* genes, have been described in *P. infestans*, including *PiAvr1* (Van der Lee et al., 2001; Ballvora et al., 2002), *PiAvr2* (Gilroy et al., 2011), *PiAvr3a* (Armstrong et al., 2005), *PiAvr3b* (Rietman et al., 2012), *PiAvr4* (Van Poppel et al., 2008), *PiAvr8* (Vossen et al., 2016), *PiAvrblb1* (Song et al., 2003; Vleeshouwers et al., 2008), *PiAvrblb2* (Van der Vossen et al., 2005; Oh et al., 2009), *PiAvrvnt1* (Foster et al., 2009; Pel et al., 2009), *PiAvrSmira1* and *PiAvrSmira2* (Rietman et al., 2012; Yoshida et al., 2013) as shown in (**Supplementary Table S1**). *P. infestans* is predicted to encode 563 RXLR effector genes which were mainly found located in repeat-rich or gene-sparse regions of the genome, meaning that more rapid evolution compared to other genes located in gene-dense regions (Haas et al., 2009; Yin et al., 2017). It might explain its ability to escape host defense mechanisms (Lenman et al., 2016). It has been well-documented that RXLR effectors play important roles in potato–*P. infestans* interactions. In rare cases of recognition by the cognate *R* genes, they mediate late blight resistance by triggering HR; in most cases, they are not recognized and function as typical virulence factors by interfering with host cell structure and function, resulting in enhancing plant susceptibility (Huang et al., 2019).

There are generally two strategies to improve late blight resistance. The first is the deployment of many different *R* genes to offer tentative durable resistance since changes of multiple effectors are predicted to increase the penalty. The second strategy is to identify genes that are capable of recognizing various effectors or core effectors. In fact, the identified *R* genes from varieties that showed durable disease resistance were confirmed to be able to recognize two or more effectors. For example, the potato *Rpi-blb1*, known as *RB* (Song et al., 2003) recognizes *PiAvrblb1*, *ipiO1* and *ipiO2* (Vleeshouwers et al., 2008; Chen et al., 2012); the *Rps1k* of soybean recognizes two *P. sojae* effectors, *Avr1b* and *Avr1k* (Shan et al., 2004; Dou et al., 2010).

Yet, *P. infestans PiAvr3a* is a well-characterized *P. infestans* RXLR effector that is highly expressed during the biotrophic phase of infection [2–3 days post in filtration (dpi)] (Haas et al., 2009; Chaparro-Garcia et al., 2015). *PiAvr3a* is essential for full virulence, pathogenicity and suppression of host immunity, including PTI and ETI, by suppressing the programmed cell death (PCD) triggered by the elicitin INF1, a secreted *P. infestans* protein with PAMP properties, by interacting with and stabilizing the host U-box E3 ligase CMPG1 (Bos et al., 2006; González-Lamothe et al., 2006; Bos et al., 2010; Gilroy et al., 2011), as well as targeting the receptor-mediated endocytosis dynamin-related protein 2B (DRP2B), clathrin-mediated endocytosis (CME) (Chaparro-Garcia et al., 2015). Two major allelic isoforms of *PiAvr3a* have been identified in *P. infestans* populations that have a difference in three amino acids in mature protein positions 19, 80 and 103 (Chapman et al., 2014). *Avr3a ^(S19)^ ^E80M103^* is known as *PiAvr3a^EM^* while *Avr3a ^(C19)^ ^K80I103^* is known as *PiAvr3a^KI^* (Vleeshouwers et al., 2011; Yang et al., 2018). Unlike *PiAvr3a^EM^*, *PiAvr3a^KI^* activates the potato *R3a* resistance protein to trigger ETI and confers avirulence to heterozygous or homozygous strains of the pathogen (Armstrong et al., 2005; Chaparro-Garcia et al., 2015). Therefore, *P. infestans* isolates, expressing only the *PiAvr3a^EM^* variant, can evade *R3a* recognition and do not trigger HR (Armstrong et al., 2005; Bos et al., 2006). Identification of natural *R* genes that can recognize *PiAvr3a^EM^*, are promising approaches to improve late blight resistance (Bos et al., 2010; Segretin et al., 2014).

*Agrobacterium tumefaciens*-mediated transient gene expression technology is a rapid, widely and easily performed assay that is commonly sed in gene expression analysis and functional genomics studies in many plant species, including *Arabidopsis thaliana*, tobacco, tomato, soybean, citrus, grapevine and potato (Vleeshouwers et al., 2008). Typically, *A. tumefaciens*-mediated transient expression assays can be utilized for several purposes, such as i) functional genomics tools for transient overexpression of a gene in planta, ii) reverse genetic studies of a gene by virus-induced gene silencing (VIGS) or RNA interference (RNAi) technology, iii) rapid accessible production of recombinant proteins, iv) pathogen effector assays for the genetic components of the selected cultivars disease resistance.

In this study, we utilized the optimum conditions for *A. tumefaciens*-mediated transient assays in potato and performed analyses of two potato varieties for their capability to recognize a set of *P. infestans* known effectors, as part of our effort in understanding late blight resistance of potato varieties that showed promising field performance. This led to the identification of natural resistance, mediated by recognition of *P. infestans Avr3a^EM^*, which will facilitate potato breeding for improved late blight resistance.

## Materials and Methods

### Plant Materials and Growth Condition

Qingshu9 and Longshu7 are dominant potato varieties in Northwestern China. Qingshu9 was derived from crosses of two parents “387521.3 × APHRODITE”, while Longshu7 was derived from Fedori×Zhuangshu3. Potato cuttings have been cultured in a sterilized MS medium for four weeks (Murashige and Skoog, 1962). Next, the germinated seedlings were transferred for another four weeks into vermiculite, and then planted in pots that contain a mix of sterilized vermiculite and peat moss (V/V = 1:2). Also, some potato differentials, including *R1*, *R2*, *R3a*, *R4* and *R8*, were used for the optimization of agroinfiltration assays. In addition, nine potato breeding lines were studied and agro-infiltrated with *PiAvr3a^EM^*. Progeny lines resulting from crossing Qingshu9 with Qingshu2, ND, NSS1-5, and Jizhang8, respectively, and Longshu7 with CIP01, CIP03, CIP16, CIP30, and CIPL06408, respectively, were evaluated. At least 20 progenies from each cross were evaluated. Potato plants were grown under standardized conditions in a greenhouse within a temperature range of 18–22°C and under a day/night regime of 16 h/8 h. Fully-expanded leaves of the 4-week old seedlings were used for infiltration with bacterial cell suspensions of *A. tumefaciens* strain AGL1 that carry a number of *Avr* genes to be evaluated.

### Cloning and Vector Construction of *P. infestans Avr* Genes

All tested *Avr* genes were amplified from their plasmid DNA previously constructed into pK7WG2 vector, using TransStart^®^ FastPfu DNA Polymerase (Applied Biosystems, USA) with *Avr* genes-specific primers containing the restriction enzyme recognition sites as shown in **Supplementary Table S2**. The PCR amplicons were purified using the TIANGEN Universal DNA Purification Kit (TianGen Biotech Co., Ltd., Beijing, China). The purified amplicons and the pART27 cloning vector were digested with the corresponding restriction enzymes and ligated together using T4 DNA ligase (Promega, USA). The ligation mixtures were transferred to *E. coli* DH5α competent cells by electroporation using standard protocols. Transformed colonies were cultured on LB medium supplemented with 100 µg ml^−1^ of spectinomycin and incubated at 37°C. Positive clones were confirmed by sequencing. The confirmed plasmid constructs were then transformed into *A. tumefaciens* strain AGL1 by the heat shock method. The transformed cell cultures were applied to LB plates containing antibiotics (100 µg ml^−1^ of spectinomycin, 20 µg ml^−1^ of rifampicin) and placed in a 28°C incubator for 2 days. A single colony was transferred using sterilized toothpicks to the liquid LB broth having the same antibiotics and incubated at 200 *rpm* in a shaker at 28°C for 2 days.

### Transient Agro-Infiltration Assays

The optimized conditions of agroinfiltration-mediated transient expression assays were, 3–4 or 9–10 week-old potato seedlings, *A. tumefaciens* strain AGL1 and OD_600_ value of 0.4. *A. tumefaciens* cells were grown in LB medium (supplemented with 50 µg ml^−1^ of gentamicin, 20 µg m l^−1^ of rifampicin and 100 µg m l^−1^ of spectinomycin, 20 µg ml^−1^ of rifampicin, respectively) up to the log phase of development. The bacterial solution was then centrifuged at room temperature (20°C, 4,000*g*, 3 min), followed by resuspension in an inducing media (10 mM MES, 200 µM acetosyringone, 10 mM MgCl_2_, pH 5.6). The optical density of the *A*. *tumefaciens* suspensions was adjusted to OD_600_ value of 0.4 and incubated before infiltration for 1–3 h at room temperature. Agroinfiltration experiments were carried out at room temperature 20 ± 2°C (Dillen et al., 1997; Su et al., 2012) on potato seedling leaves 4-week-old and the results were scored from 3 dpi and typically photographed at 5–7 dpi.

### Optimization of Agro-Infiltration Assays

To evaluate factors affecting the agro-infiltration assay, three parameters were assessed, including different *A. tumefaciens* strains (AGL1 and GV3101), bacterial cell densities (OD_600_ values of 0.2, 0.3, 0.4, 0.5, and 0.6), and different growth ages of cultured potato plants (3–4, 6–7 and 9–10 week-old). All experiments have been repeated three times with 20 replicates for each. In our study, potato seedling age refers to the time starting from tissue culture seedlings transferred to the soil matrix, after acclimatization in the vermiculite, to the time of experimentation.

### Effector Screen

Ten *P. infestans Avr* effectors were investigated including, *PiAvr1*, *PiAvr2*, *PiAvr3a^KI^*, *PiAvr3b*, *PiAvr4*, *PiAvrblb1*, *PiAvrblb2*, *PiAvrsmira1*, *PiAvr8* (*PiAvrsmira2*) and *PiAvrvnt1.1* (**Supplementary Table S1**). *A. tumefaciens* strain AGL1 carrying each of these effectors was used for infiltration in the two varieties with a concentration of OD_600_ value of 0.4. Further investigation was done for *PiAvr3a* alleles, *PiAvr3a^EM^* and *PiAvr3a^KI^*. Forty leaves were agro-infiltrated for each *Avr* effector in eight independent experiments with five replicates for each. All pictures were taken 5–7 days later of infiltration.

### Detection of *R8* and *Rpi-vnt1* by PCR Amplification

Genomic DNA was isolated from fresh leaf tissue of both potato varieties using the CTAB-based protocol. The genomic DNA was then subjected to PCR with primers specific to *R8* and *Rpi-vnt1* (Foster et al., 2009; Pel et al., 2009; Vossen et al., 2016), as listed in the **Supplementary Table S3**. PCR reactions were performed using FastPfu DNA polymerase (Applied Biosystems, USA). Each PCR reaction contained 30 μl PCR mix, including 6 μl 5× FastPfu Buffer, 2.4 μl dNTPs (0.2 mM), 1 μl total genomic DNA (100 ng), 0.2 μl MgSO_4_ (50mM), 0.6 μl each forward and reverse primers (0.2 mM), 0.6 μl FastPfu DNA polymerase (2.5 units) and 18.6 μl dH_2_O. The PCR amplification was carried out by denaturing at 95°C for 2 min, followed by 40 cycles of 94°C for 20 s, 55°C for 20 s and 72°C for 1 min, and a final extension time of 5 min at 72°C. PCR products were separated by gel electrophoresis on a 1% agarose gel and DNA bands were visualized under UV on the Quantum CX5 Imaging System.

### *P. infestans* Infection Assays on Detached Potato Leaves

*P. infestans* isolates were cultured and maintained on a rye sucrose agar (RSA) medium. All plates were then grown at 16°C in darkness for two weeks. The sporangial suspensions were prepared by washing and rubbing the culture with 5 ml distilled water. Then, the sporangial suspension concentration was adjusted to 4 × 10^4^ sporangia/ml before cooled down for 2 h at 4°C to promote release of motile zoospores for inoculation (Tian et al., 2015). Leaflets of 6 –10 week-old potato plants were placed abaxially on plastic trays on a filter paper saturated with dH_2_O. All leaflets were drop-inoculated with 15 μl sporangial/zoospore suspension on the abaxial side. Six *P. infestans* isolates, PjY009, PjY048, PjY061, Pa21106, Pd21410 and F48, were used in the inoculation assays (**Supplementary Figure S2**). Inoculation with dH_2_O was considered as a control treatment. All plastic trays were covered by a plastic wrap and incubated in a growth chamber at 16–18°C with >75% relative humidity in the darkness in order to ensure infection. Results were recorded as a lesion diameter of the inoculated area were and pictures were taken five days after inoculation. Disease resistance or susceptibility were recorded by using a scale reported for disease severity (Sun, 2012).

## Results

### Optimization of *A. tumefaciens*-Mediated Transient Gene Expression Assay

**Figure 1:**
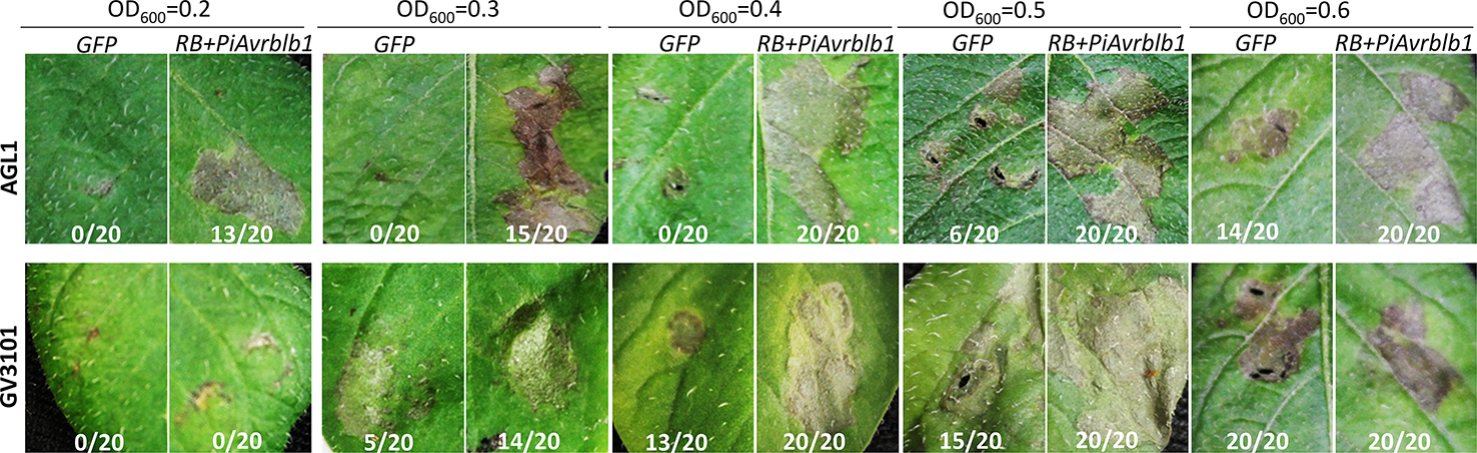
Specific hypersensitive response (HR) mediated by recognition of *P. infestans Avr* genes in potato is affected by strains used for *A. tumefaciens* infiltration. Strains AGL1 and GV3101 were examined with different optical densities, OD_600_ of 0.2, 0.3, 0.4, 0.5 and 0.6, in the potato differential genotype *R3*. Co-infiltration of agrobacteria carrying *PiAvrblb1* and *RB* was used as a positive treatment, while an *A. tumefaciens* carrying Green Fluorescent Protein gene (*GFP*) was infiltrated as a negative control. The number of HR sites/total number of infiltration sites were indicated. The experiment was repeated three times with 20 replicates. Pictures were taken at 5–7 days dpi.

The outcome of plant-*Agrobacterium* interactions is determined by the genetic background of both partners. In addition to the efficiency of transient gene expression, the frequent non-specific necrotic response is a major concern in the use of this assay. We therefore examined for suitable *A. tumefaciens* strains with reduced background necrotic reaction in potato. Six different *A. tumefaciens* strains, Agro-1D124g, GV3101, AGL1, 1100, LBA4404, and EHA105, were evaluated on ten different potato varieties (Data not shown). Even though the OD_600_ value was very low, strains 1100, LBA4404 and EHA105 induced a high rate of background necrosis on most of the potato cultivars. However, with a lower concentration of bacterial suspensions, GV3101 and AGL1 strains showed a significant reduction of background reaction on most potato varieties. Thus, strains GV3101 and AGL1 were employed to further investigate their transient expression efficiencies on the potato. The efficiency assay was examined by the HR symptoms that resulted from the co-infiltration of *P. infestans Avr* gene *PiAvrblb1* and its cognate resistance gene *RB*. The results showed that the AGL1 strain was more efficient than GV3101 in terms of triggering specific HR mediated by co-expression of *RB* and *PiAvrblb1* (**Figure 1**).

We also evaluated various potato seedling growth ages (3–4, 6–7 and 9–10 week-old) for the effect on the efficiency of the agro-infiltration assay. Five potato differential lines, including *R1*, *R2*, *R3a*, *R4*, and *R8*, were examined and agro-infiltrated with *A. tumefaciens* AGL1 suspensions (OD_600_ of 0.4) carrying the *P. infestans Avr* genes *PiAvr1*, *PiAvr2*, *PiAvr3a^KI^*, *PiAvr4*, and *PiAvr8*, respectively. The positive control was the co-infiltration of mixed agrobacteria carrying *PiAvrblb1* and *RB* which would lead to HR while the negative control was the *GFP*. The most consistent and efficient infiltration was observed while using terminal leaflets from 3–4 and 9–10 week-old potato plants in all tested differential lines, while the 6–7 week potato leaves exhibited less efficient transient expression (**Supplementary Figure S1**). We speculated that the potato leaves were younger in 3–4 weeks when the leaves have just spread and the main veins were developed, but the lateral veins were not obvious, allowing easier infiltration in whole leaves. Meanwhile, the leaves of 9–10 week-old seedlings were fully developed, and the main and lateral veins were well developed, allowing efficient infiltration between the two lateral veins. However, the main veins and lateral veins of leaves of 6–7 week-old seedlings were all developed, still small interveinal spaces on the abaxial side hinder the infiltration process, making the bacterial solution restricted to a fixed grid, necessitating more infiltration sites. The optimum condition was utilized for further analysis which could be summarized as using the *A. tumefaciens* strain AGL1, with an OD_600_ value of 0.4 and leaves of the 3–4 or 9–10 week-old seedlings.

**Figure 2:**
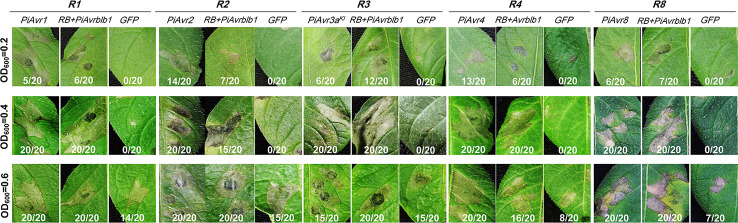
Effects of OD_600_ values of agrobacterial suspension on the HR response mediated by recognition of *P. infestans Avr* genes. Different OD_600_ values, 0.2, 0.4 and 0.6, were examined for genotype-specific HR triggered by *P. infestans Avr* genes on potato differential lines carrying the cognate *R* genes. *A. tumefaciens* AGL1 suspensions carrying *P. infestans Avr* genes *PiAvr1*, *PiAvr2*, *PiAvr3a^KI^*, *PiAvr4*, and *PiAvr8*, were infiltrated in potato differential genotypes carrying *R1*, *R2*, *R3a*, *R4* and *R8*, respectively. Co-infiltration of agrobacteria carrying *PiAvrblb1* and *RB* was used as a positive control, while *GFP* as a negative control. The number of HR sites/total number of infiltration sites were indicated. The experiment was repeated three times with 20 replicates. All images were taken at 5–7 dpi.

To further confirm our improved agroinfiltration assay, we examined known *Avr* effector genes for their capability in triggering genotype-specific HR. *A. tumefaciens* AGL1 bacterial suspensions carrying the *P. infestans Avr* genes *PiAvr1*, *PiAvr2*, *PiAvr3a^KI^*, *PiAvr4*, and *PiAvr8* were infiltrated in potato differential lines carrying genotype-specific *R* genes *R1*, *R2*, *R3a*, *R4* and *R8*, respectively. Each density (OD_600_ values of 0.2, 0.4 and 0.6) showed a different level of transient expression. The bacterial suspensions with OD_600_ value of 0.4 consistently displayed the highest efficiency in bacterial infiltration assays, as all tested *Avr* genes induced genotype-specific HR in all tested differential lines (**Figure 2**). While at a higher agrobacterial concentration of OD_600_ of 0.6, an increase of HR response for all tested *Avr* genes and significant background necrosis for the negative control of *GFP* expression were observed in all tested differentials, though at the lower agrobacterial concentration (OD_600_ of 0.2) the HR triggered by *PiAvr3a* and *PiAvr8* in potato differential lines *R3a* and *R8*, respectively, were not visible.

### Evaluation of Two Potato Varieties for Recognizing Known *P. infestans Avr* Genes

**Figure 3:**
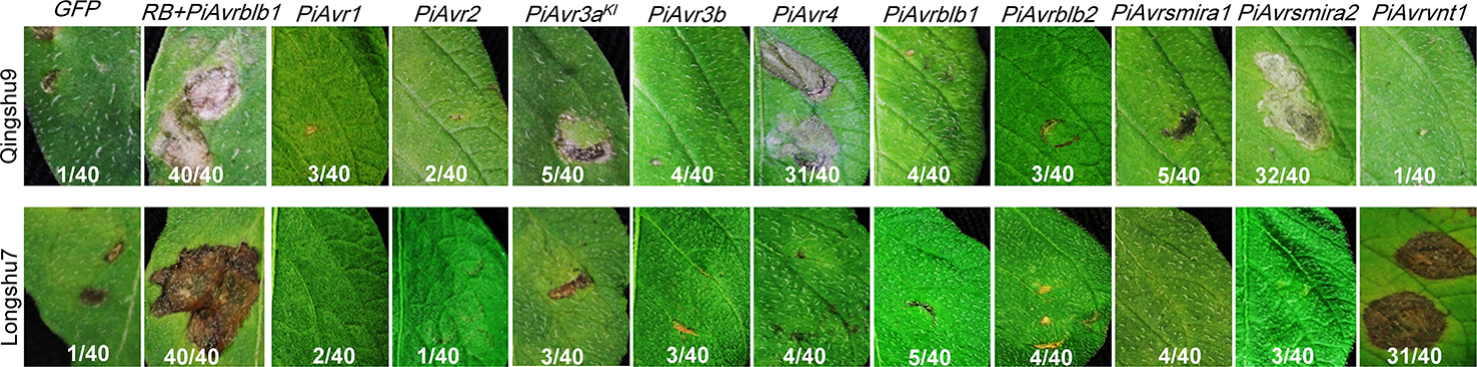
Specific HR induced by 10 known *P. infestans Avr* genes in two potato varieties, Qingshu9 and Longshu7. In Qingshu9, the typical HR was induced by *PiAvr4* and *PiAvrsmira2*, whereas Longshu7 was responsive to *PiAvrvnt1.1. A. tumefaciens* AGL1 suspension with an OD_600_ value of 0.4 was used for infiltration of 4-week-old potato leaves. *GFP* was used as the negative control, shown at the left side, while the positive control was indicated by co-infiltration of agrobacteria carrying *PiAvrblb1* and *RB*, respectively. The indicated are the number of HR-responsive leaves/total number of the infiltrated leaves. All pictures were taken at 5–7 dpi.

To understand late blight resistance of two Chinese potato varieties, Qingshu9 and Longshu7, that showed excellent field performance with a low percentage of disease incidence and severity (Wang et al., 2018), we evaluated whether they contain known *R* genes by examining their capability to recognize corresponding 10 *P. infestans Avr* genes. Both Qingshu9 and Longshu7 showed typical genotype-specific HR phenotypes 5 days post infiltration with *A. tumefaciens* AGL1 cell suspensions with an OD_600_ of 0.4. Qingshu9 showed HR triggered by two *P. infestans Avr* genes, *PiAvr4* and *PiAvr8*, suggesting the presence of *R* genes *R4* and *R8*. Longshu7 showed HR triggered by *PiAvrvnt1.1*, indicating the existence of *Rpi-vnt1* (**Figure 3**). Furthermore, the presence of *R8* and *Rpi-vnt1* in Qingshu9 and Longshu7, respectively, was preliminarily analyzed by PCR amplification using gene-specific primers (Foster et al., 2009; Pel et al., 2009; Vossen et al., 2016) (**Supplementary Figure S3**). However, whether they are functional *R* genes needs further validation. PCR amplification might provide possibility for their presence since it is highly dependent on the specific primers, while the potential presence of functional *R* gene homologs/alleles may lead to false negative results. The agroinfiltration assay using effector genes is an efficient method to detect the presence of functional *R* genes, such as *R8* and *Rpi-vnt1.1* in this research.

### Qingshu9 and Longshu7 Showed Genotype-Specific HR Triggered by *Avr3a^EM^*

**Figure 4:**
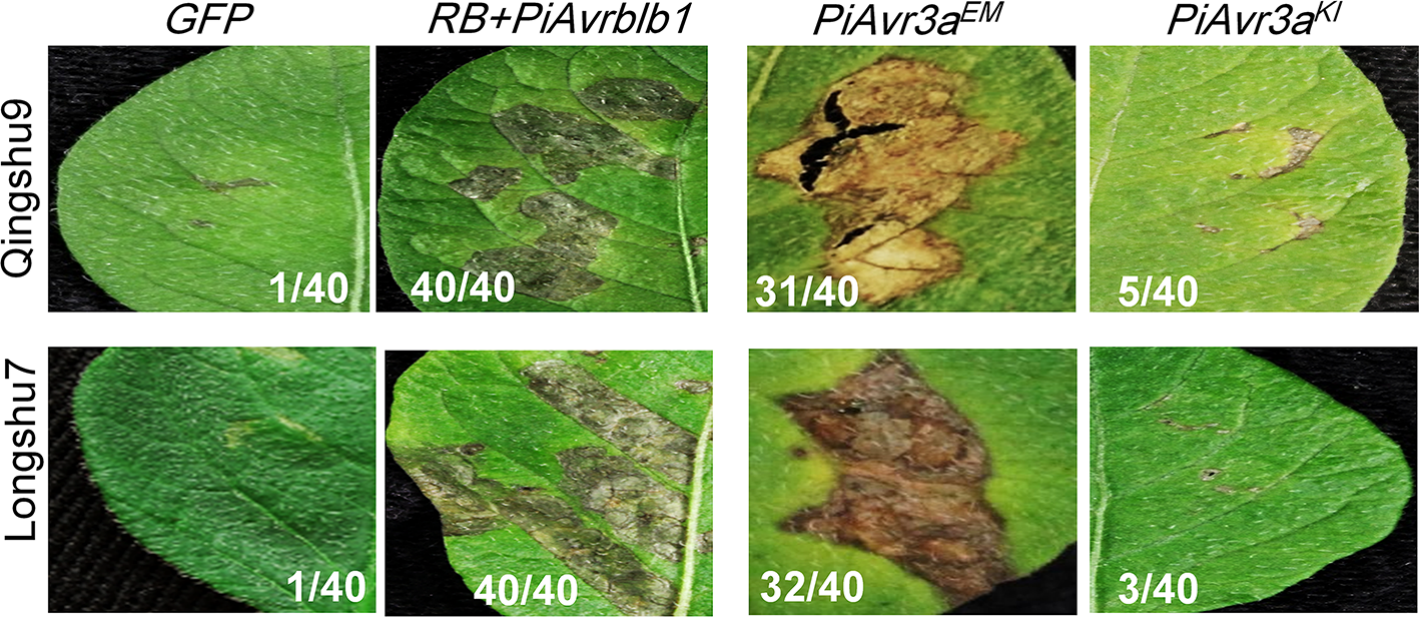
Potato varieties Qingshu9 and Longshu7 were capable of recognizing *P. infestans PiAvr3a^EM^*. Both varieties were triggered with HR when infiltrated with agrobacteria carrying *PiAvr3a^EM^*, a virulent allele of *PiAvr3a^KI^*, but not by *PiAvr3a^KI^*. The *A. tumefaciens* AGL1 bacterial suspension with an OD_600_ of 0.4 was used for infiltration of the 4-week-old potato leaves. The HR positive control was indicated by co-expression of *PiAvrblb1* and *RB*, shown at the left side, and *GFP* was used as the negative control. The number of HR-responsive leaves/total number of the infiltrated leaves, were indicated. All pictures were taken at 5–7 dpi.

*P. infestans Avr* gene *PiAvr3a^KI^* can be specifically recognized by the cognate *R3a*. However, the number of its virulent alleles, that escaped recognition by *R3a*, is very limited and the virulent allele *PiAvr3a^EM^* is widely present around the world, suggesting the vital role of *PiAvr3a* in *P. infestans* pathogenesis. The identification of varieties with capable *PiAvr3a^EM^* recognition that make it possible for breeding new varieties with the capability to recognize both *PiAvr3a^KI^* and *PiAvr3a^EM^*, which is predicted to improve durable resistance against late blight. Both Qingshu9 and Longshu7 showed an HR upon *PiAvr3a^EM^* infiltration, but not upon *PiAvr3a^KI^* (**Figure 4**).

### Recognition of *P. infestans Avr3a^EM^* by Qingshu9 and Longshu7 Is Likely Conferred by a Single Gene

**Figure 5:**
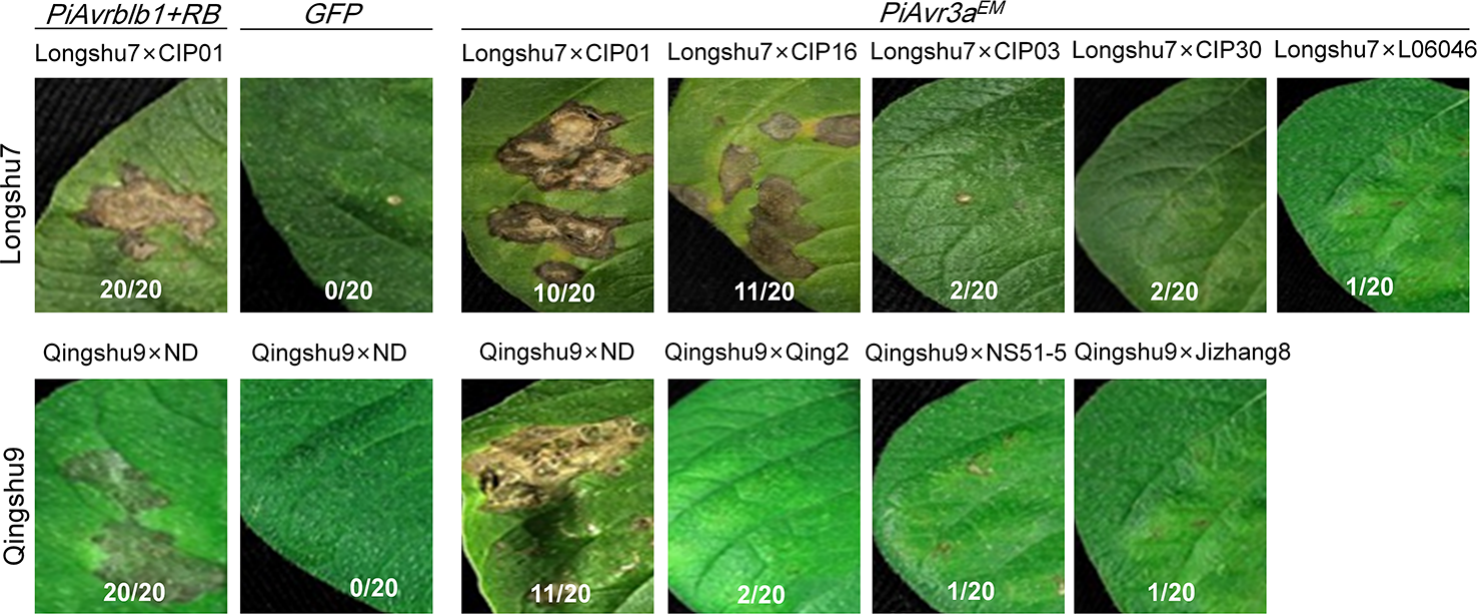
Segregation of HR induced by recognition of *P. infestans PiAvr3a^EM^* in F1 populations using Longshu7 or Qingshu9 as a parental. Progenies derived from crosses Longshu7 X CIP01 and Longshu7 X CIP16 showed typical HR as that in Longshu7, and progenies from cross Qingshu9 X ND showed typical HR as that in Qingshu9. *GFP* was used as negative control and co-expression of *PiAvrblb1* and *RB* was used as a positive control. The number of HR-responsive progenies/total number of progenies, were indicated in each crosses, with each progenies examined with 30 infiltration sites. All pictures were taken at 5–7 dpi.

Given the fact that *PiAvr3a^KI^* (Armstrong et al., 2005) is recognized by *R3a* (Huang et al., 2005), a cloned and well-studied *R* gene, we predict that *PiAvr3a^EM^* is similarly recognized by a single *R* gene. We therefore employed several independent F1 segregation populations to ensure that the *PiAvr3a^EM^* recognition is conditioned by a single *R* gene, by examining whether the *PiAvr3a^EM^* recognition-triggered cell death segregates. We performed agroinfiltration assays for progenies derived from a total of nine crosses for the two responsive varieties, with five crosses using Longshu7 as a resistant parental with five non-responsive potato clones as the susceptible parental, including CIP01, CIP03, CIP16, CIP30, and CIPL06408. Qingshu9 as the resistant parental was crossed with four non-responsive potato clones as the susceptible parental, including Qingshu2, ND, NSS1-5, and Jizhang8. Twenty F1 progenies from each cross were tested for their response upon infiltration with *PiAvr3a^EM^*, with a total of 30 infiltration sites for each progenies. The results showed that progenies derived from two investigated Longshu7 crosses showed an HR response upon *PiAvr3a^EM^* infiltration at a rate of 1:1 for each cross, including Longshu7 X CIP01 and Longshu7 X CIP16 as shown in (**Figure 5**) and supplementary (**Supplementary Table S4**). While for Qingshu9 crosses, progenies from only one investigated cross (Qingshu9 X ND) showed an HR response with a rate of 1:1 (**Figure 5**, **Supplementary Table S4**). Although we did not perform comprehensive genetic analysis, the segregation of *PiAvr3a^EM^* recognition strongly suggests that *PiAvr3a^EM^* recognition by Qingshu9 and Longshu7 is conditioned by a single *R* gene. Also, the results suggest that the *R* genes for *PiAvr3a^EM^* recognition in Qingshu9 and Longshu7 were heterozygous, and most, if not all, parental lines crossed with Qingshu9 or Longshu7 were unable to recognize *PiAvr3a^EM^*. Another possibility is that a helper/sensor NLR might be required for *R3a* function to initiate the immune signaling, resulting in an HR response, similar to the case of NRC4, a helper NLR essential for immunity triggered by *Rpi-blb2* (Wu et al., 2017).

## Discussion

A critical step in the successful agroinfiltration-mediated transient expression is the establishment of harmonious interaction between the plant and *A. tumefaciens*. We also considered potential background non-specific necrotic reactions frequently caused by molecules from *A. tumefaciens*. We examined multiple *A. tumefaciens* strains (Agro-1D124g, GV3101, AGL1, 1100, LBA4404, and EHA105), potato genotypes, growth stages, and bacterial densities. This led to the conclusion that AGL1 was the most efficient strain with fewer background effects than GV3101, which is consistent with a report showing that strain AGL1 was preferred for potato whereas GV3101 was more suitable for *Nicotiana benthamiana* (Du et al., 2014).

Potato leaves at various growth stages were also a major concern and we found that the maximum level of bacterial infiltration was observed at the terminal leaflets from 3–4 to 9–10 week-old potato seedlings. The efficiency of infiltration became much lower when leaves were used from 6–7 week-old seedlings. Changes in the expression levels may be related to general changes in leaf physiology, especially soluble protein concentration during leaf aging (Halfhill et al., 2003; Wydro et al., 2006). Also leaf morphological characteristics including, leaf surface, the thickness of the cuticle layer and epidermis, stomatal size and frequency, veins, trichomes, the midrib structure, and interveinal distribution on the abaxial side leaves may affect infiltration efficiency (Abdullah and Halterman, 2018). Some developmental ages have a low density of trichomes on the abaxial side and the leaf veins on the surface are not prominent and as a result, facilitate infiltration (as 4-week-old leaves). Besides, the older leaves (9–10-week-old) where interveinal space has increased showed an increase in transient expression. Meanwhile, the 6–7-week-old leaves exhibited an irregular leaf surface with a high density of trichomes on the abaxial side, prominent veins and small interveinal spaces that can hinder the infiltration process.

Ten *Avr* genes had been described in *P. infestans* and their gain-of-virulence alleles were reported (Vleeshouwers and Oliver, 2014). In this study, we aimed at detecting and identifying *R* genes in potato varieties that showed excellent performance against late blight. Agroinfiltration analysis of 10 *P. infestans Avr* genes was done using *A. tumefaciens* strain AGL1 with OD_600_ value of 0.4 to evaluate two potato varieties, Qingshu9 and Longshu7, whether they encode *R* genes that may recognize these *Avr* genes. The agroinfiltration in Qingshu9 resulted in activating a typical HR towards three effector genes including, *PiAvr4*, *PiAvr8* and *PiAvr3a^EM^*, which is a virulent allele of *PiAvr3a* that can be recognized by *R3a*, whereas Longshu7 exhibited a typical HR by two *P. infestans* effector genes, *PiAvr3a^EM^* and *PiAvrvnt1*. According to the gene-for-gene hypothesis, *Avr* genes are detected by its counterpart *R* genes (Anderson et al., 2015; Yin et al., 2017). These results suggest that Qingshu9 may carry at least three *R* genes, including *R4*, *R8*, and R3a^*^, whereas Longshu7 carries at least two *R* genes, *Rpi*-*vnt1* and R3a^*^.

*PiAvr3a* was highlighted and has been extensively studied which is expected to be a useful target for potato breeders seeking durable resistance (Cooke et al., 2012). *PiAvr3a* appears to be a core effector of *P. infestans* since it's among few effectors that are conserved across several *Phytophthora* species and it is consistently induced within the early stages of *P. infestans* infection (Yin et al., 2017). Besides, it is involved in the suppression of PTI and ETI (Gilroy et al., 2011; Franco-Orozco et al., 2017). So far, there are only two detected *PiAvr3a* alleles among *P. infestans* populations (Bos et al., 2010). The avirulent allele, *PiAvr3a^KI^*, is recognized by *R3a*, while its virulent allele, *PiAvr3a^EM^*, evades recognition by *R3a* (Armstrong et al., 2005; Chapman et al., 2014).

Previous studies showed successful recognition of *PiAvr3a^EM^* by engineering potato resistance gene *R3a* (Chapman et al., 2014) and by screening the *R3a* variants library resulting from random mutagenesis of the full-length *R3a* coding sequence (Segretin et al., 2014). Remarkably, our results offer a new natural resistance gene that can recognize *PiAvr3a^EM^* in two potato varieties, suggesting that these two varieties are potentially undergoing R3a^*^-mediated recognition responses. Both varieties were derived from crosses that used different parents, for Longshu7 being derived from Fedori×Zhuangshu3, while Qingshu9 from 387521.3 × APHRODITE, suggesting that both varieties might contain a functional homolog of the R3a* resistance gene and both are very likely heterozygous. It's also possible that they might have two different forms of R3a* that mediate *PiAvr3^EM^* recognition. Both varieties didn't show any *PiAvr3^KI^* recognition, suggesting that they don't carry the known *R3a*.

Further work on the survey of *PiAvr3^EM^* -mediated HR on progenies derived from crosses using either Qingshu9 or Longshu7 as a parental indicated that progenies from two Longshu7 crosses were detected with *PiAvr3a^EM^*-mediated HR, while progenies from a single Qingshu9 cross were detected for inducing an HR response, suggesting that the recognition of *PiAvr3a^EM^* is most likely conditioned by a single *R* gene R3a^*^ in both varieties. Most lines that were crossed with either Longshu7 or Qingshu9, if not all, do not carry R3a^*^. The lack of *PiAvr3a^EM^* response in some populations is likely resulted from the heterologous nature of *PiAvr3a^EM^* recognition in the resistant parents and short of *PiAvr3a^EM^* recognition in the other parents. It's also possible that we examined limited number of progenies. However, whether the *PiAvr3a^EM^* recognition in Longshu7 or Qingshu9 mediates late blight resistance needs additional pathogenicity tests. Under favorable infection conditions using detached leaves, our preliminary infection assays with diverse virulent *P. infestans* strains showed generally high levels of late blight resistance for Longshu7 and Qingshu9, though certain level of susceptibility was notable to several virulent strains (**Supplementary Figure S2**). There are potentially complicated interactions between effectors in suppression and triggering immune response. A promising efficient strategy to enhance late blight resistance is to integrate *R3a* that recognizes *PiAvr3a^KI^* and R3a* that mediates *PiAvr3a^EM^* response. However, whether such simple *R* gene combination is correlated with predicted enhanced durable late blight resistance needs confirmation by field assessments.

*Rpi-Smira2* (*R8*) confers quantitative resistance under field conditions and associates with *PiAvrSmira2* (*PiAvr8*) (Rietman et al., 2012; Hajianfar et al., 2014). In our study, Qingshu9 exhibited an HR response upon *PiAvr8*/*PiAvrSmira2* infiltration, suggesting the presence of *R8*/*Rpi-smira2* in Qingshu9. The *PiAvr8*/*PiAvrSmira2*-triggered HR in Qingshu9 was consistent with a previous report in which *R8* is correlated with quantitative resistance and *PiAvr8*/*PiAvrSmira2* triggered *R8*-mediated resistance (Rietman et al., 2012). Our findings are also consistent with a report in which genotype-specific HR was induced after *R8*-*PiAvr8* co-infiltration as well as *R8*-like co-infiltration with *PiAvr8* (Jiang et al., 2018). Notably, the NB-LRR gene *R8* has been cloned and was thought to provide broad-spectrum and durable field resistance against *P. infestans* (Vossen et al., 2016; Jiang et al., 2018). It has been reported that *Rpi-Smira2* co-localized with the *R8* locus and both loci conferred similar resistance levels (Jo, 2013; Stefańczyk et al., 2017). Hence, it was suggested that *Rpi-Smira2* and *R8* are identical or functional homologs (Jo et al., 2011). In addition, many *P. infestans* isolates carry *PiAvr8* that was reported to trigger an HR response of the *R8* gene in disease resistant potato varieties and lines, such as Sarpo Mira from Europe, PB-06, S-60, and QTL dPI09c from China, and Jacqueline Lee from USA (Vossen et al., 2016; Jiang et al., 2018), suggesting its vital role in the pathogen and the effectiveness of *R8*.

*PiAvrvnt1* is recognized by the potato resistance gene *Rpi-phu1*/*Rpi-vnt1* (Foster et al., 2009). Because of its polymorphism, it is associated with a response to a diversified target protein or recognition avoidance (Pel et al., 2009; Pais et al., 2017). Longshu7 showed HR toward *Avrvnt1.1*, suggesting that it carries the functional *Rpi-vnt1* gene which may provide a high level and wide-spectrum late blight resistance (Stefańczyk et al., 2018).

In summary, we developed and used the optimized *A. tumefaciens*-mediated transient expression assays to evaluate two potato varieties Qingshu9 and Longshu7 that showed years of promising field late blight resistance for *R* genes they might carry, by detecting the presence of HR triggered by 10 known *P. infestans Avr* genes. This led to the identification of natural resistance mediated by recognition of *PiAvr3a^EM^*, a globally present virulence allele of *PiAvr3a^KI^* that plays vital roles in potato-*P. infestans* interactions. Interestingly, cloning and analysis of R3a^*^ that mediates *PiAvr3a^EM^* recognition and other detected *R* genes in Qingshu9 and Longshu7 will be interesting to make both good use of late blight resistance and improved understanding of disease resistance in future. Together with the identification of additional complementary *R* genes in the two varieties, these findings will facilitate the development of potato lines with a high level of late blight resistance, by pyramiding these promising *R* genes.

## Data Availability Statement

The datasets generated for this study are available on request to the corresponding author.

## Author Contributions

WS and YM designed the experiments. AE, JL, XW, CZ, and YM performed the experiments. AE, JL, YM, GW, JW, HL-K, and WS analyzed the data. AE, YM, and WS wrote the manuscript with contribution from all authors.

## Funding

This work was supported by National Natural Science Foundation of China (31561143007), China Agriculture Research System (CARS-09), Potato Breeding Program from Department of Science and Technology of Ningxia (#2019NYYZ01), and the Programme of Introducing Talents of Innovative Discipline to Universities (project 111) from the State Administration of Foreign Experts Affairs (#B18042).

## Conflict of Interest

The authors declare that the research was conducted in the absence of any commercial or financial relationships that could be construed as a potential conflict of interest.

## Supplementary Material

Supplementary Table S1 List of *Avr* genes of late blight pathogen *P. infestans* with their corresponding *R* genes.

Supplementary Table S2 List of all specific primers used for cloning *P. infestans Avr* genes.

Supplementary Table S3 List of specific primers used for detection of *R8* and *Rpi-vnt1* by PCR amplification.

Supplementary Table S4 Segregation for *PiAvr3a^EM^* response in the F1 populations of crosses Longshu7 X CIP01, Longshu7 X CIP16, and Qingshu9 X ND.

Supplementary Figure S1 Effect of various growth ages of potato seedlings on the efficiency of *Agrobacterium tumefaciens*-mediated transient expression. Leaves of three different growth stages, 3-4, 6-7 and 9-10 week-old seedlings, were investigated. Five *P. infestans Avr* genes *PiAvr1, PiAvr2, PiAvr3a^KI^, PiAvr4* and *PiAvr8* were expressed by *A. tumefaciens* AGL1, OD_600_ of 0.4, in five potato differential genotypes carrying cognate *R* genes *R1*, *R2*, *R3a*, *R4* and *R8*, respectively. Co-infiltration of *PiAvrblb1* and *RB* was used as a positive control, whereas *GFP* used as a negative control. The number of HR sites/total number of infiltration sites were indicated in each treatment. All photographs were taken 5 dpi.

Supplementary Figure S2 Infection of Qingshu9 and Longshu7 to different *P. infestans* strains. Detached leaflets of 9-10 week-old plants were drop-inoculated with 4×10^4^ sporangia/mL of six *P. infestans* strains with known virulence spectrum, including PjY009, PjY048, PjY061 (Yunnan, Southwestern China), Pa21106, Pd21410 (Ningxia, Northwestern China) and F48 (Fujian, Southeastern China), with sterilized dH_2_O as the control treatment. Qingshu9 was highly resistant (R) to Pa21106, Pd21410 and F48, but susceptible (S) to PjY009, PjY048 and PjY061. Longshu7 was resistant to PjY061, Pa21106, Pd21410 and F48, but susceptible to PjY009 and PjY048. Disease index (DI) were scored and photographs were taken 5 dpi. The experiment was repeated four timed with five replicates.

Supplementary Figure S3 Detection of *R8* and *Rpi-vnt1* in Qingshu9 and Longshu7 by PCR amplification using gene-specific primers. Two sets of gene-specific primers for *R8* and *Rpi-vnt1* each were used for their detection.
