# Correction: Identification of natural resistance mediated by recognition of *Phytophthora infestans* effector gene *Avr3a^EM^* in potato

**DOI:** 10.3389/fpls.2026.1919638

**Published:** 2026-07-16

**Authors:** Ahmed S. M. Elnahal, Jinyang Li, Xiaoxia Wang, Chenyao Zhou, Guohong Wen, Jian Wang, Hannele Lindqvist-Kreuze, Yuling Meng, Weixing Shan

**Affiliations:** 1 State Key Laboratory of Crop Stress Biology for Arid Areas and College of Plant Protection, Northwest A&F University, Yangling, China; 2 Plant Pathology Department, Faculty of Agriculture, Zagazig University, Zagazig, Egypt; 3 Institute of Potato Research, Gansu Academy of Agricultural Sciences, Lanzhou, China; 4 Institute of Biotechnology, Qinghai Academy of Agricultural Sciences, Xining, China; 5 International Potato Center, Lima, Peru; 6 State Key Laboratory of Crop Stress Biology for Arid Areas and College of Agronomy, Northwest A&F University, Yangling, China

**Keywords:** potato late blight, *Phytophthora infestans*, Qingshu9, Longshu7, RXLR effectors, hypersensitive response

There was a mistake in **Figure 5** as published. While preparing the composite **Figure 5**, the same image was inadvertently placed in the affected panel of the *PiAvr3a^EM^* section to illustrate the frequent absence of cell death responses in potato progenies. In addition, the GFP negative-control panel in the published figure missed its corresponding label. In the corrected **Figure 5**, the missing GFP label has been restored, and the affected *PiAvr3a^EM^* panel has been replaced with the appropriate available original image corresponding to the correct progeny. These errors were confined to the preparation of the figure assembly and do not affect the underlying interpretation of the experiment or the conclusions drawn from the figure.

The corrected **Figure 5** appears below:

**Figure 5:**
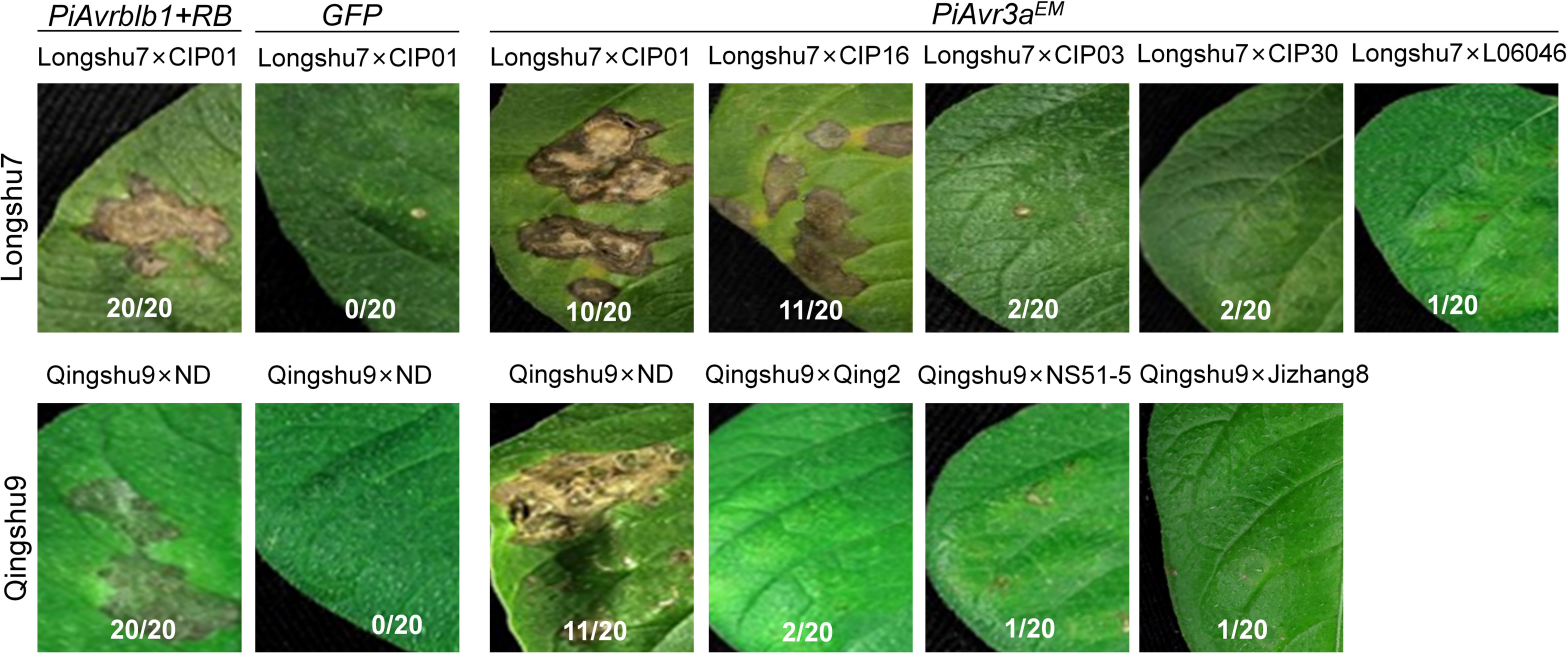
Segregation of HR induced by recognition of P. infestans PiAvr3aEM in F1 populations using Longshu7 or Qingshu9 as a parental. Progenies derived from crosses Longshu7 X CIP01 and Longshu7 X CIP16 showed typical HR as that in Longshu7, and progenies from cross Qingshu9 X ND showed typical HR as that in Qingshu9. GFP was used as negative control and co-expression of PiAvrblb1 and RB was used as a positive control. The number of HR-responsive progenies/total number of progenies, were indicated in each crosses, with each progenies examined with 30 infiltration sites. All pictures were taken at 5–7 dpi.

The original version of this article has been updated.

