# Supplementary material for: Identification of Natural Resistance Mediated by Recognition of Phytophthora infestans Effector Gene Avr3aEM in Potato
Source: Front Plant Sci. 2020 Jun 19;11:919. doi: 10.3389/fpls.2020.00919 (PMC7318898; doi:10.3389/fpls.2020.00919)
Supplement: Supplementary Table S1 — List of Avr genes of late blight pathogen P. infestans with their corresponding R genes. [file DataSheet_1.docx]

**Supplementary Tables**

**Supplementary Table S1:** List of *Avr* genes of late blight pathogen *P. infestans* with their corresponding *R* gene.

| **Gene ID** | **Known *Avr* Gene** | **RxLR** | ***R* gene** | **References** |
| --- | --- | --- | --- | --- |
| PITG_16663 | *Avr1* | RQLR | *R1* | Ballvora et al. 2002; Tyler 2009 |
| PITG_22870 | *Avr2* | RLLR | *R2* | Gilroy et al. 2011; Lokossou et al. 2009;  Saunders et al. 2012 |
| PITG_14371 | *Avr3a* | RLLR | *R3a* | Armstrong et al. 2005; Bos et al. 2010;  Engelhardt et al. 2012; Huang et al. 2005 |
| PITG_18215 | *Avr3b* | RSLR | *R3b* | Jiang et al. 2006; Li et al. 2011;  Rietman 2011; van der Lee et al. 2001 |
| PITG_07387 | *Avr4* | RFLR | *R4* | van Poppel et al. 2008 |
| PITG_21388 | *Avrblb1* | RSLR | *Rpi*-*blb1* | Champouret et al. 2009; Chen et al. 2012;  Song et al. 2003; van der Vossen et al. 2003;  Vleeshouwers et al. 2008 |
| PITG_20300 | *Avrblb2* | RSLR | *Rpi*-*blb2* | Bozkurt et al. 2011; Oh et al. 2009;  van der Vossen et al. 2005 |
| PITG_07550 | *Avrsmira1* | RLLR | *Rpi*-*Smira1* | Rietman et al. 2012 |
| PITG_07558 | *Avrsmira2/Avr8* | RSLR | *Rpi*-*Smira2*/*R8* | Jo 2013; Jo et al. 2011;  Rietman et al. 2012 |
| PITG_16294 | *Avrvnt1* | RLLR | *Rpi*-*vnt1** | Foster et al. 2009; Pel et al. 2009;  Vleeshouwers et al. 2011 |

**Supplementary Table S2:** List of all specific primers used for cloning the *P. infestans* *Avr* genes.

| **Primer name** | **Primer(5'-3')** |
| --- | --- |
| *Avr1*-F | GGAATTCATGGCCGGATTCGACCACG |
| *Avr1*-R | CCCAAGCTTTTAAAATGGTACCACAACATGTCCA |
| *Avr2*-F | CCGCTCGAGATGCTGCATGCAGCTCCAGG |
| *Avr2*-R | GCTCTAGATTAACTCCTCTTGTCACCCTTAATT |
| *Avr3a^KI^*-F | CCGCTCGAGCATGCGTCTGGCAATTATGCTGT |
| *Avr3a^KI^*-R | GCTCTAGACTAATATCCAGTGAGCCCCAGG |
| *Avr3a^EM^*-F | CCGCTCGAGATGATCGACCAAACCAAGGTCCT |
| *Avr3a^EM^*-R | GCTCTAGACTAATATCCAGTGAGCCCCAGG |
| *Avr3b*-F | CCGCTCGAGATGACGTACTCGACTTCAAAGG |
| *Avr3b*-R | GCTCTAGATTAGAAATTGTTCTTTGCGGTCAG |
| *Avr4*-F | CCGCTCGAGATGGATTCTTTAGCTCGTACCGT |
| *Avr4*-R | GCTCTAGACTAAGATATGGGCCGTCTAGCT |
| *Avrblb1*-F | CCGCTCGAGATGAATCTCAACACCGCCGTG |
| *Avrblb1*-R | GCTCTAGACTAGCTAGGGCCAACGTTTTTAT |
| *Avrblb2*-F | CCGCTCGAGATGTTCCCAATCCCCGACGA |
| *Avrblb2*-R | GCTCTAGATCAGGACTTCGTCATTTTTGCTT |
| *Avrsmira1*-F | CCGCTCGAGATGGCAACTGTGACCAAGGTATC |
| *Avrsmira1*-R | GCTCTAGATTATCCGGAGGGGTTTAGCGAG |
| *Avrsmira2*-F | CCGCTCGAGATGACACCAGCACCGCCAC |
| *Avrsmira2*-R | GCTCTAGATTACGATGTTTTCGCTTCTTTAAAAAGC |
| *Avrvnt1*-F | CCGCTCGAGATGGTAACGACCCCGACCAA |
| *Avrvnt1*-R | GCTCTAGATCAAGCTCTAATAGGATCAAGCT |

Underline represent restriction sites: XhoI site (CTCGAG), XbaI site (TCTAGA), EcoRI site (GAATTC). ClaI site (ATCGAT), and HindIII site (AAGCTT).

**Supplementary Table S3:** List of specific primers used for *R8* and *Rpi-vnt1* detection by the molecular PCR test.

| **Gene name** | **Marker** | **Primer orientation** | **Primer sequence (5ʹ-3ʹ)** | **Annealing temperature**  **(°C)** | **size (bp)** |
| --- | --- | --- | --- | --- | --- |
| *Rpi-vnt1* | vntNBSHae **^a^** | Forward | CTTACTTTCCCTTCCTCATCCTCAC | 60 | 555 |
|  |  | Reverse | TGAAGTCATCTTCCAGACCGATG | 60 |  |
|  | NBS3B **^b^** | Forward | CCTTCCTCATCCTCACATTTAG | 65 | 612 |
|  |  | Reverse | GCATGCCAACTATTGAAACAAC | 65 |  |
| *R8* | RGA3.1-1 **^c^** | Forward | CACCTAACTGATTTGCTTC | 58.4 | 1806 |
|  |  | Reverse | GTATGTGCACCCCATGATGA | 58.4 |  |
|  | RGA3.1-2 **^c^** | Forward | TCATCATGGGGTGCACATAC | 58.4 | 1904 |
|  |  | Reverse | TGGATTGAGCTTTCTGGATTC | 58.4 |  |

Abbreviations: bp = base pare, **^a^** (Foster *et al.* 2009)^,^ **^b^** Pel *et al.* 2009, **^c^** (Vossen *et al.* 2016),

**Supplementary Table S4:** Segregation for *PiAvr3a^EM^* response in the F1 populations of crosses Longshu7 X CIP01, Longshu7 X CIP16, and Qingshu9 X ND.

| Longshu7 X CIP01 | | | | | Longshu7 X CIP16 | | | | | Qingshu9 X ND | | | | |
| --- | --- | --- | --- | --- | --- | --- | --- | --- | --- | --- | --- | --- | --- | --- |
| Progeny clone | *Avr3a^EM^* | *GFP* | *RB*+ *Avrblb1* | No of HR sites**^a^** | Progeny clone | *Avr3a^EM^* | *GFP* | *RB*+ *Avrblb1* | No of HR sites**^a^** | Progeny clone | *Avr3a^EM^* | GFP | *RB*+ *Avrblb1* | No of HR sites**^a^** |
| 1 | + | - | + | 24 | 1 | + | - | + | 25 | 1 | + | - | + | 22 |
| 2 | - | - | + | 3 | 2 | + | - | + | 23 | 2 | - | - | + | 3 |
| 3 | + | - | + | 23 | 3 | + | - | + | 24 | 3 | + | - | + | 24 |
| 4 | + | - | + | 22 | 4 | - | - | + | 4 | 4 | - | - | + | 5 |
| 5 | - | - | + | 5 | 5 | - | - | + | 2 | 5 | + | - | + | 23 |
| 6 | + | - | + | 25 | 6 | - | - | + | 5 | 6 | - | - | + | 3 |
| 7 | - | - | + | 4 | 7 | + | - | + | 22 | 7 | + | - | + | 22 |
| 8 | + | - | + | 24 | 8 | + | - | + | 24 | 8 | - | - | + | 4 |
| 9 | - | - | + | 2 | 9 | - | - | + | 5 | 9 | + | - | + | 24 |
| 10 | - | - | + | 4 | 10 | + | - | + | 23 | 10 | - | - | + | 3 |
| 11 | + | - | + | 23 | 11 | - | - | + | 4 | 11 | + | - | + | 23 |
| 12 | + | - | + | 22 | 12 | + | - | + | 23 | 12 | - | - | + | 4 |
| 13 | - | - | + | 3 | 13 | - | - | + | 3 | 13 | + | - | + | 24 |
| 14 | - | - | + | 5 | 14 | + | - | + | 22 | 14 | + | - | + | 22 |
| 15 | + | - | + | 24 | 15 | + | - | + | 24 | 15 | + | - | + | 23 |
| 16 | - | - | + | 1 | 16 | - | - | + | 5 | 16 | - | - | + | 2 |
| 17 | + | - | + | 22 | 17 | - | - | + | 5 | 17 | - | - | + | 3 |
| 18 | - | - | + | 4 | 18 | - | - | + | 3 | 18 | - | - | + | 5 |
| 19 | + | - | + | 25 | 19 | + | - | + | 24 | 19 | + | - | + | 22 |
| 20 | - | - | + | 3 | 20 | + | - | + | 22 | 20 | + | - | + | 23 |

**^a^** No of HR sites from a total of 30 infiltration sites.
