# Supplementary figures and images for: Identification of Natural Resistance Mediated by Recognition of Phytophthora infestans Effector Gene Avr3aEM in Potato
Source: Front Plant Sci. 2020 Jun 19;11:919. doi: 10.3389/fpls.2020.00919 (PMC7318898; doi:10.3389/fpls.2020.00919)

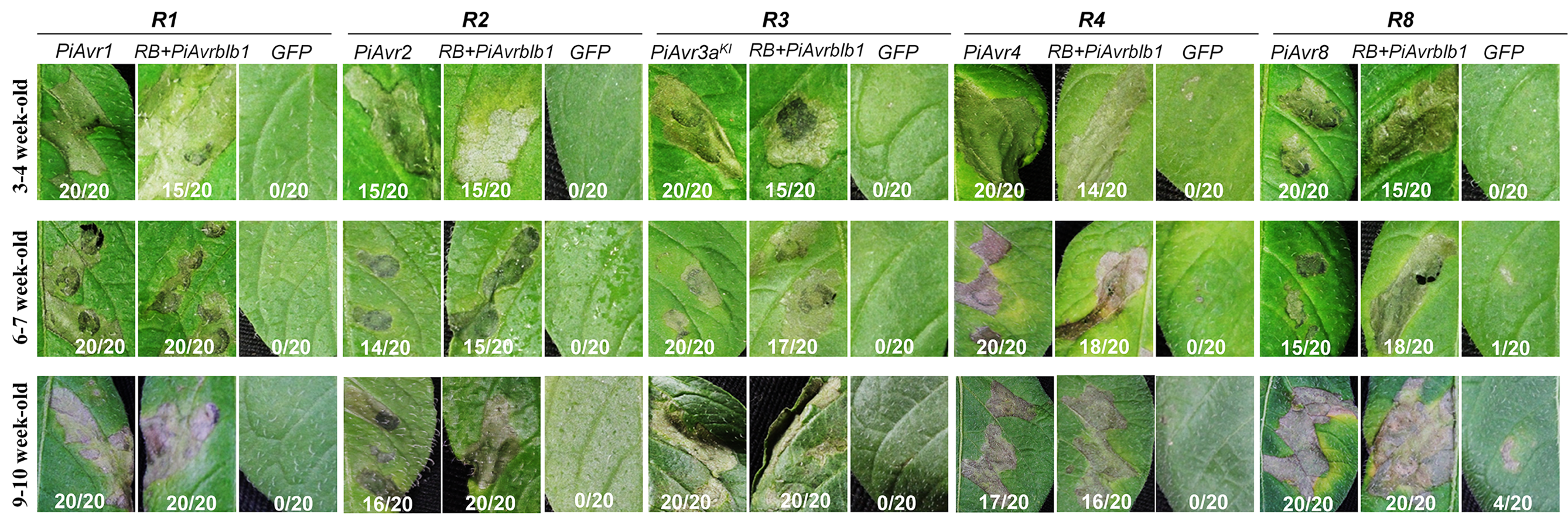

Supplement: Supplementary Figure S1 — Effect of various growth ages of potato seedlings on the efficiency of Agrobacterium tumefaciens-mediated transient expression. Leaves of three different growth stages, 3-4, 6-7 and 9-10 week-old seedlings, were investigated. Five P. infestans Avr genes PiAvr1, PiAvr2, PiAvr3aKI, PiAvr4 and PiAvr8 were expressed by A. tumefaciens AGL1, OD600 of 0.4, in five potato differential genotypes carrying cognate R genes R1, R2, R3a, R4 and R8, respectively. Co-infiltration of PiAvrblb1 and RB was used as a positive control, whereas GFP used as a negative control. The number of HR sites/total number of infiltration sites were indicated in each treatment. All photographs were taken 5 dpi. [file Image_1.tif]

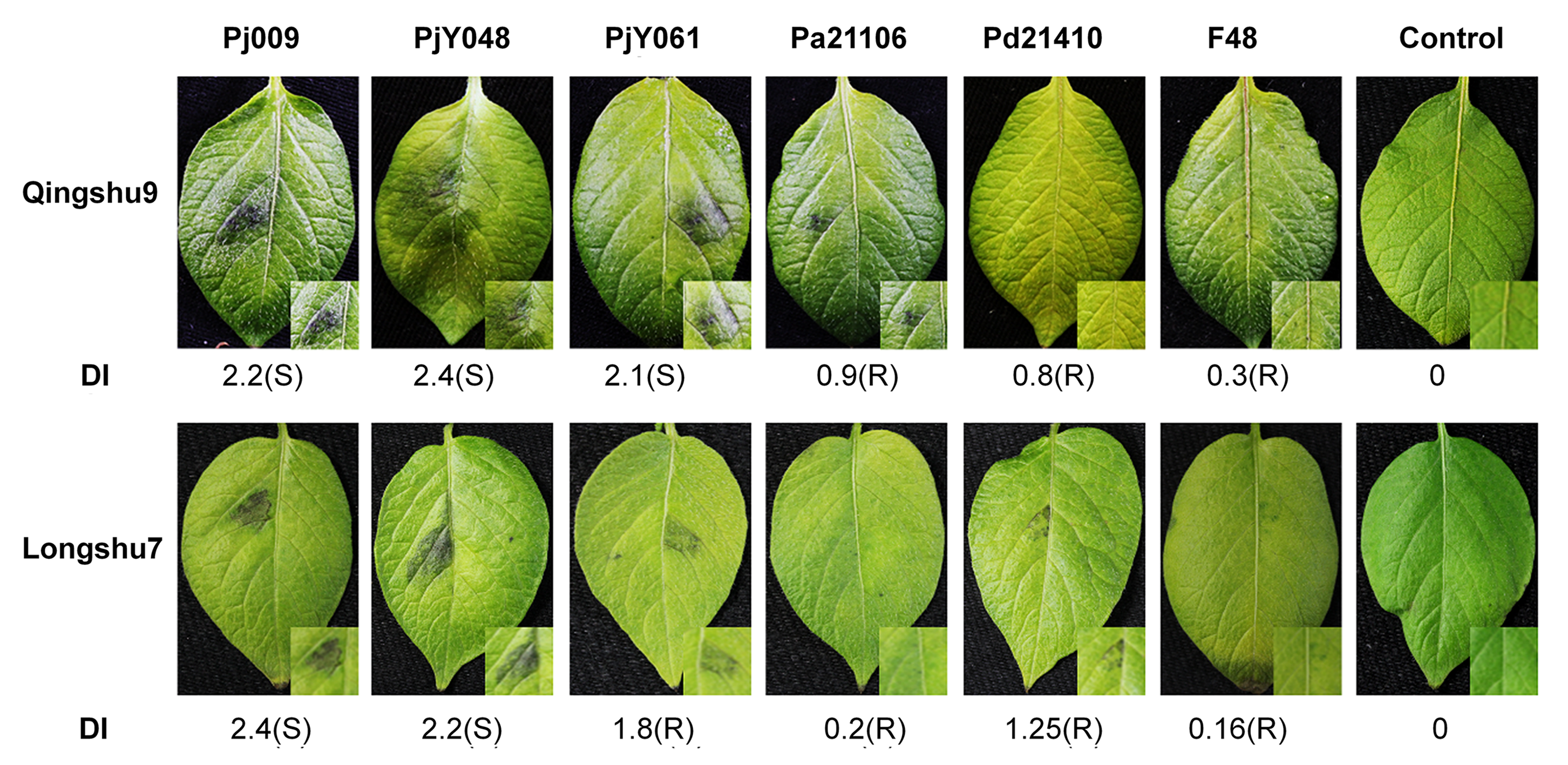

Supplement: Supplementary Figure S2 — Infection of Qingshu9 and Longshu7 to different P. infestans strains. Detached leaflets of 9-10 week-old plants were drop-inoculated with 4×10^4^ sporangia/mL of six P. infestans strains with known virulence spectrum, including PjY009, PjY048, PjY061 (Yunnan, Southwestern China), Pa21106, Pd21410 (Ningxia, Northwestern China) and F48 (Fujian, Southeastern China), with sterilized dH2O as the control treatment. Qingshu9 was highly resistant (R) to Pa21106, Pd21410 and F48, but susceptible (S) to PjY009, PjY048 and PjY061. Longshu7 was resistant to PjY061, Pa21106, Pd21410 and F48, but susceptible to PjY009 and PjY048. Disease index (DI) were scored and photographs were taken 5 dpi. The experiment was repeated four timed with five replicates. [file Image_2.tif]

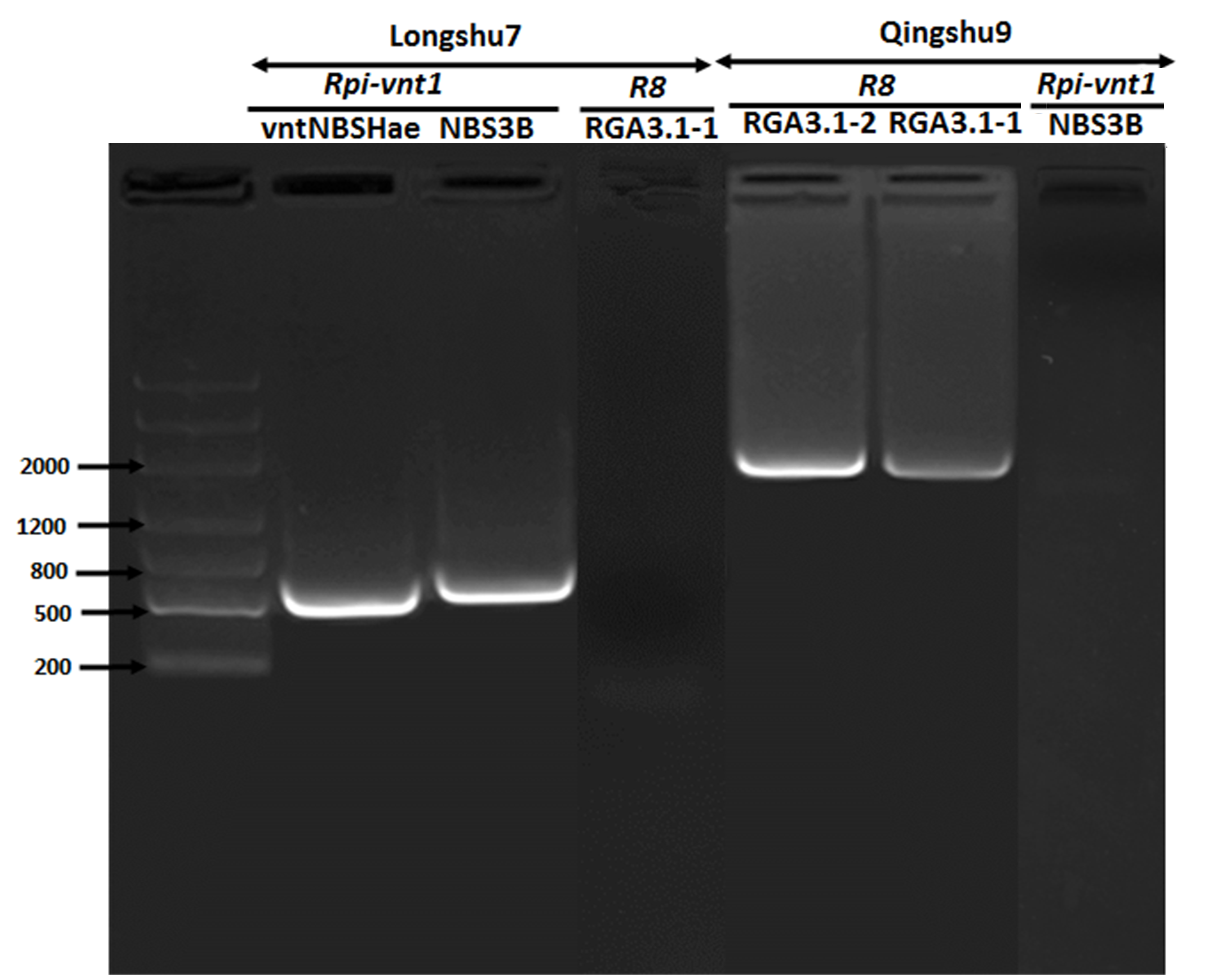

Supplement: Supplementary Figure S3 — Detection of R8 and Rpi-vnt1 in Qingshu9 and Longshu7 by PCR amplification using gene-specific primers. Two sets of gene-specific primers for R8 and Rpi-vnt1 each were used for their detection. [file Image_3.tif]
